# RNA-seq-based digital gene expression analysis reveals modification of host defense responses by rice stripe virus during disease symptom development in Arabidopsis

**DOI:** 10.1186/s12985-016-0663-7

**Published:** 2016-12-02

**Authors:** Feng Sun, Peng Fang, Juan Li, Linlin Du, Ying Lan, Tong Zhou, Yongjian Fan, Wenbiao Shen, Yijun Zhou

**Affiliations:** 1 Institute of Plant Protection, Jiangsu Academy of Agricultural Sciences; Jiangsu Technical Service Center of Diagnosis and Detection for Plant Virus Diseases, Nanjing, 210014 China; 2 College of Life Science, Nanjing Agricultural University, Nanjing, 210095 China

**Keywords:** Rice stripe virus, RNA-seq, Digital gene expression (DGE), Defense response

## Abstract

**Background:**

Virus infection induces and suppresses host gene expression on a global level. Rice stripe virus (RSV) is the type species of the genus *Tenuivirus* and infects rice and Arabidopsis plants. Microarray-based and next generation sequencing-based transcriptomic approaches have been used to study rice-RSV interactions. However, our knowledge of the response of Arabidopsis plants to RSV infection is limited, and it requires further investigation to determine the similarities (or differences) in virus-host interactions between monocot and dicot hosts infected with RSV.

**Methods:**

We characterized transcriptome changes in *Arabidopsis thaliana* infected with rice stripe virus (RSV) with RNA-seq based digital gene expression (DGE) analysis. The transcriptomes of RSV-infected samples were compared to those of mock-treated samples at 14 and 21 days post-infection (dpi) during different stages of symptom development.

**Results:**

We identified 624 differentially expressed genes (DEGs) in Arabidopsis influenced by RSV at 14 dpi and 21 dpi, among which at 14 dpi, 255 transcripts were induced, and 38 were repressed; at 21 dpi, 146 were induced, and 237 were repressed. Functional annotation indicated that these DEGs were related to multiple biological functions, including defense response, secondary metabolism, protein amino acid phosphorylation and response to abiotic stress.

**Conclusions:**

Importantly, the transcription of genes related to host defense systems was activated by RSV infection at an early stage of symptom development (14 dpi), whereas over the infection period (21 dpi), the host defense response systems were suppressed. A total of 52 genes were continuously differentially expressed between the two time points, indicating that the majority of DEGs were transient and unique to a particular time point during symptom development. The DEGs, particularly the defense response genes, identified in this study are candidates suitable for further functional analysis during the RSV-Arabidopsis interaction.

**Electronic supplementary material:**

The online version of this article (doi:10.1186/s12985-016-0663-7) contains supplementary material, which is available to authorized users.

## Background

In host plants, viruses can manipulate host metabolites for translation and replication of their genomes and silence host responses by suppressors [1–3]. The interplay between the host plant and the invading virus causes host cells to up- or down-regulate certain pathways, inducing host plant physiological and phenotypic changes, which suggests the involvement of numerous host genes [4–6]. One main task of plant virologists is to understand the mechanisms underlying plant-virus interactions. To achieve this, transcriptome profiling has been adopted to reveal how a virus colonizes a host, how a host mounts a defense response against a virus, and how a compatible virus-host interaction results in disease symptoms.

Rice stripe virus (RSV) is the type species of the genus *Tenuivirus* and primarily infects rice plants [7, 8]. RSV is transmitted transovarially in a circulative manner by vector insects, primarily the small brown planthopper (SBPH; *Laodelphax striatellus* Fallen) [9, 10]. The genome of RSV consist of four single-stranded RNA segments, containing seven open reading frames (ORFs). RNA1 has negative polarity and encodes a protein of 337 kDa, which is a putative viral RNA-dependent RNA polymerase (RdRp) [11]. The three smaller RNA segments (RNAs 2, 3 and 4) are ambisense [12, 13], each contain two ORFs which encode proteins associated with functions including virus movement, encapsidation, RNA silencing suppression, transcription, and planthopper transmission [8, 14, 15].

In nature, RSV can infect rice plants and cause severe rice stripe disease; in the laboratory, RSV can infect *Nicotiana benthamiana* through mechanical inoculation and *Arabidopsis thaliana* through viruliferous insect inoculation [14, 16]. Rice and Arabidopsis plants infected with RSV all show similar disease symptoms, including yellow stripes on leaves, severe stunting and even death [16]. To understand the mechanism of plants responses to RSV infection and identify important genes involved in plant-RSV interactions, microarray-based and next generation sequencing-based transcriptomic approaches have been used to study rice-RSV interactions. Microarray analysis indicates that RSV infection selectively modifies the transcription of rice genes related to protein-synthesis, energy production, cell structure and defense systems depending on the viral titer and symptom development [17]. Furthermore, RNA-Seq analysis demonstrates that in RSV-infected rice plants, down-regulation of chloroplast genes is associated with disease symptom development [18, 19] and host defense pathways are selectively suppressed by RSV in both susceptible and resistant rice cultivars [19, 20]. Small RNA deep sequencing analysis showed that RSV infection induces the accumulation of novel or phased siRNAs or miRNAs and selectively modifies the expression of a conserved miRNA family [19, 21]. However, our knowledge of the response of Arabidopsis plants to RSV infection is limited, and it requires further investigation to determine the similarities (or differences) in virus-host interactions between monocot and dicot hosts infected with RSV.

To characterize Arabidopsis responses to RSV infection at the transcriptome level, we performed a temporal transcriptome analysis across 2 time points for up to 21 dpi to identify co-regulated defense and stress mechanisms activated (or suppressed) by RSV. Time-course gene-expression analysis in Arabidopsis infected with RSV indicated that during early stages of symptom development (14 dpi), RSV induced plant defense responses but this response was repressed at later stage of symptom development (21 dpi) when the virus had accumulated. Thus, timely expression changes of genes involved in defense responses may facilitate RSV propagation and induce symptoms in Arabidopsis. Altogether, this study provides insights that contribute to the understanding of the mechanisms underlying dicot hosts-RSV interactions.

## Methods

### Sources of virus, vectors and plant materials

Rice plants infected with RSV were collected from Jiangsu province in China. Young instar nymphs of SBPHs were fed on the RSV-infected rice plants for 2 days to acquire the virus and were maintained on “wuyujing No. 3” rice plants grown in an insect-rearing room at a temperature of 25 ± 3 °C, 55 ± 5% RH and under a light intensity of 200 μmol m^−2^ s^−1^ (14 h photoperiod). Viruliferous SBPHs were confirmed by dot-ELISA [16].

*Arabidopsis thaliana* (ecotype Columbia-0, Col-0) seeds were grown in potting soil in a growth chamber at 24 °C under 200 μmol m^−2^ s^−1^ illumination and 16-h light ⁄ 8-h dark photoperiod conditions.

### RSV inoculation assay

*Arabidopsis thaliana* plants were inoculated with 10 viruliferous SBPHs per plant and were kept in a growth chamber containing ten plants. After incubation for 4 days, planthoppers were removed. Plants were maintained in a growth chamber for symptom development, RSV-free SBPHs were used for mock inoculation.

### ELISA

Arabidopsis plants (0.1 g) were ground in liquid nitrogen and suspended with 500 μl 0.02 mol/L phosphate buffered saline (PBS). The extract was centrifuged for 3 min at 8000 × g and the supernatant was 10 fold diluted with PBS buffer and load into wells (100 μl/well) of ELISA microplants. After incubation 1 h at 37 °C, wells contained crude extracts were blocked with 1 h with 5% milk in PBST buffer. After washing, the wells were incubated with anti-RSV antibody for 1 h at 37 °C and followed by incubated with the goat anti-rabbit IgG/HRP conjugate for 1 h at 37 °C. The signals were developed in tetramethylbenzidine substrate (Sigma) and the absorbance at OD 450 was measured with a Microplant Reader Model 680 (BIO-RAD, Hercules, CA, USA).

### Western blotting

To determine RSV CP protein accumulation in Arabidopsis plants, RSV-infected Arabidopsis total proteins were extracted from 0.1 g of ground plant material in 200 μl of 2 × SDS-loading buffer. For protein gel blot, proteins were run in a 12% SDS-PAGE and transferred to PVDF membranes (BioRad, Hercules, CA, USA). The membranes were blocked for 1 h with 5% milk in PBST buffer at room temperature. After washing, the membranes were incubated with anti-RSV antibody or anti-actin antibody (Enogene, Nanjing, China) overnight at 4 °C. Signals were developed in ECL buffer (Transgen Biotech, Beijing, China) and recorded with a FUSION-SOLO2 chemical luminescence imaging system (VILBER, France).

### Illumina sequencing

Total RNA was extracted from Arabidopsis inoculated with or without RSV using Trizol reagent (Invitrogen), according to the manufacturer’s instructions. mRNA was purified from total RNA with oligo (dT) magnetic beads, then the first- and second-strand cDNAs were synthesized using oligo (dT) primers. 5′ cDNAs were digested with *Nla*III and were ligated with Illumina adaptor 1. The 3′ cDNAs were enriched using oligo (dT) magnetic beads and were ligated with Illumina adaptor 2 after removal of the magnetic beads. After 15 cycles of PCR with Illumina adaptor 1 and 2 primers, the amplified cDNA libraries were sequenced with an Illumina HiSeq 2000.

### Sequence analysis and identification of DEGs

The raw sequence data of four samples in this test have been uploaded to NCBI (http://trace.ncbi.nlm.nih.gov/Traces/sra) with the following accession numbers (SRR4034845, SRR4034846, SRR4034847, SRR4034848). The original data from Illumina sequencing were raw reads, and the clean reads were obtained after removing adaptor sequences and low quality reads. All clean reads were mapped to Arabidopsis reference sequences (TAIR 10) using bowtie software and allowing a 2-bp mismatch. Each gene’s expression level was calculated using reads per kilobase per million mapped reads (RPKM). Differentially expressed genes were identified by a *p* value ≤ 0.05 and an expression change of 2-fold or more (|log2Foldchange| ≥ 1) between the two samples using IDEG6 software [22].

### Functional annotation of DEGs

Each DEG was functionally classified based on the Arabidopsis MIPS (Munich Information Centre for Protein Sequence, http://mips.helmholtz- muenchen.de/funcatDB/) classification scheme [23] and The Arabidopsis Information Resource (TAIR 10). All DEGs were categorized using the Gene Ontology (GO) framework using the Database for Annotation, Visualization and Integrated Discovery (DAVID) v6 [24] and singular enrichment analysis (SEA) was performed with the agriGO tool [25] with default settings. A *P*-value cut-off of 0.05 was used to determine enriched GO pathways. A heat map was built using a hierarchical average linkage clustering algorithm and Pearson correlation distance metric, with the GeneSpring v. 7.3 software.

### Quantitative reverse-transcription PCR (qRT-PCR)

Total RNA was isolated from leaves using the RNAiso Plus reagent (TAKARA, Dalian, China), according to the manufacturer^’^s instructions. Arabidopsis cDNA was synthesized from 1 μg of total RNA in a volume of 20 μl using the iScript^™^ cDNA Synthesis Kit (BioRad, Hercules, CA, USA) according to the manufacturer’s instructions. qRT-PCR was performed using the SsoFast EvaGreen Supermix (BioRad, Hercules, CA, USA) with the Bio-Rad iQ5 Real-Time PCR system with gene specific primers (Additional file 1), each reaction containing 10 μl SsoFast EvaGreen Supermix, 1 μl cDNA, 1 μl primers and 8 μl water. The expression levels of transcripts are presented relative to the corresponding control samples for each condition, *EF1-a* and *actin2* were used as internal control gene [26, 27].

## Results

### Symptom development and virus accumulation in RSV-infected Arabidopsis

**Fig. 1:**
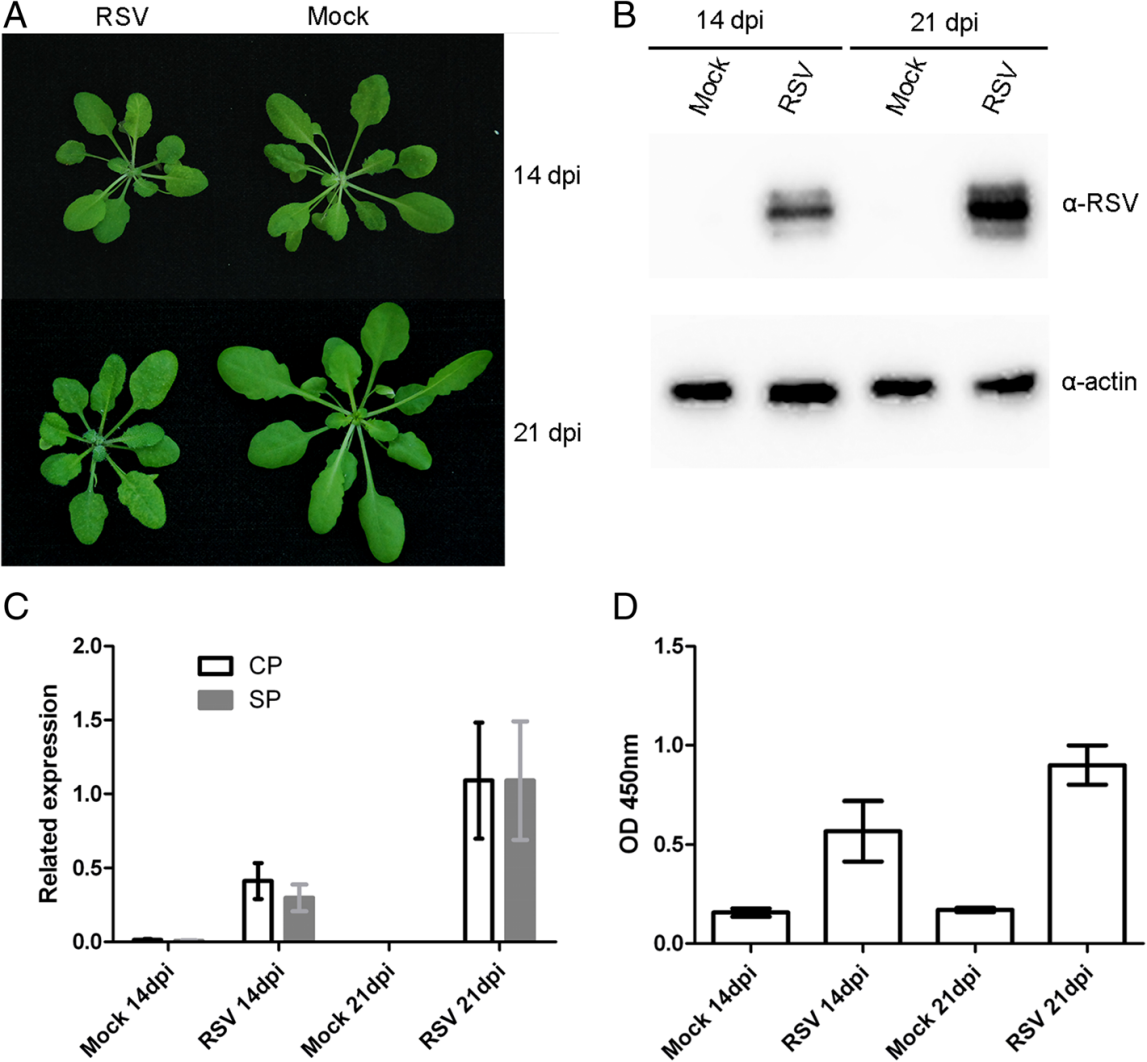
Rice stripe virus (RSV) infection in *Arabidopsis thaliana*. **a** The *left panel* shows symptom of *A. thaliana* plants inoculated with RSV, and the *right panel* shows the mock-inoculated plants. **b** RSV accumulation was estimated in Arabidopsis plants using Western blotting with a RSV specific antibody. The actin protein level served as a loading control. **c** qRT-PCR for expression of RSV CP and SP genes in infected Arabidopsis plants. Signal intensities for each transcript were normalized with *EF1-α* and *actin2.* **d** Accumulation of RSV titer in infected Arabidopsis plants by ELISA

Four-week-old Arabidopsis plants (ecotype: Col-0) were inoculated with RSV viruliferous SBPHs, and mock plants were inoculated with virus-free SBPHs (mock). Symptoms of chlorotic stripe on newly emerged leaves started to appear as early as 14 days post-inoculation (dpi). Most infected plants had significantly stunted growth and vein chlorosis on leaves at 21 dpi. (Fig. 1a). RSV accumulation in inoculated *A. thaliana* plants at 14 and 21 dpi were measured by Western blotting, qRT-PCR and ELISA. We found what the RSV titer in Arabidopsis plants increased significantly over time (Fig. 1b, c, d) and was associated with plant disease symptom development.

### RNA- seq analysis of Arabidopsis inoculated with RSV

To investigate the transcriptional responses of the Arabidopsis plants to RSV, RNA from three plants from each treatment were mixed to construct 4 cDNA libraries (RSV-14 dpi, RSV-21 dpi, Mock-14 dpi, Mock-21 dpi, Fig. 1) for RNA-seq analysis on an Illumina HiSeq 2000 platform. After adaptor sequence trimming and removing low quality reads, clean reads were obtained from four libraries of “RSV” and “Mock” samples (Table 1). Clean reads were mapped to the Arabidopsis reference genome (TAIR10, www.arabidopsis.org) using bowtie software and allowing for a 2-bp mismatch. The results are shown in Table 1, over 90% of the clean reads per library could be mapped to the reference database and the proportion of mapped gene numbers to reference gene numbers exceeded 77% in these four libraries (Table 1). These results indicated that our RNA-seq data were sufficient for subsequent gene expression analysis.

**Table 1:** Summary of sequencing data

Sample	Clean Reads	Reads mapped to genome	Mapped Rate (%)	Mapped gene numbers	Mapped gene Rate (%)
RSV-14 dpi	11,808,200	11,265,471	95.4	22,960	80.9
Mock-14 dpi	10,939,572	10,629,525	97.2	22,726	80.0
RSV-21 dpi	9,221,544	8,643,636	93.7	22,279	78.5
Mock-21 dpi	9,512,677	9,169,993	96.4	22,050	77.7

### Identification of differentially expressed genes (DEGs) in RSV-infected Arabidopsis

**Fig. 2:**
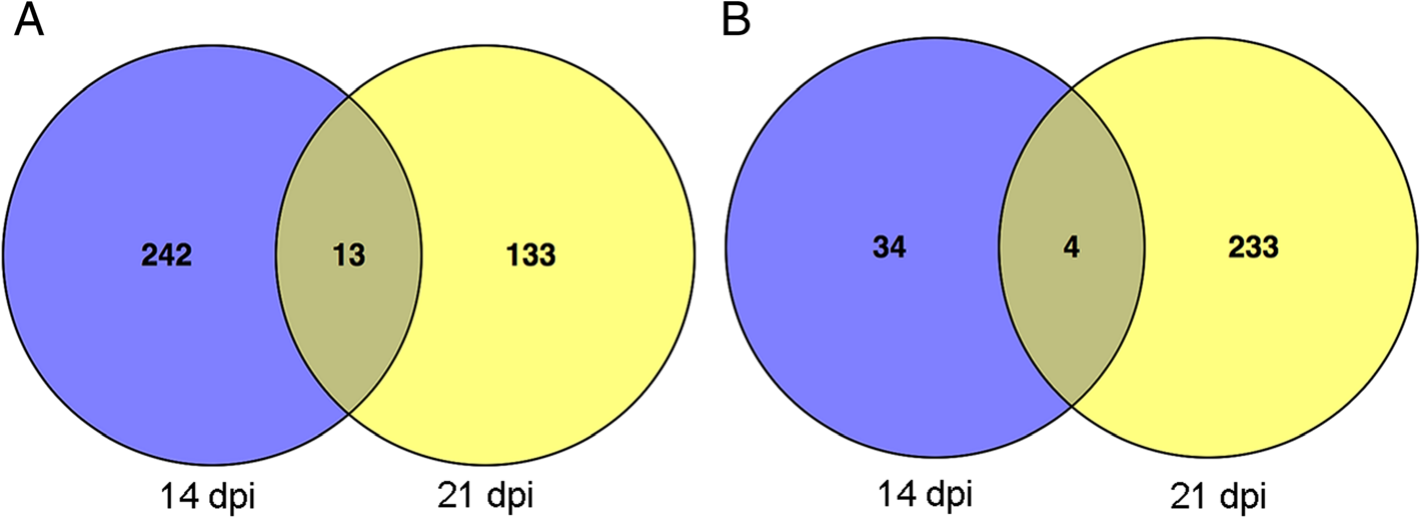
Venn diagram depicting the distribution of 624 differentially expressed genes (*p*<0.05) in RSV-infected leaf tissue at two time points post infection. **a** 388 induced transcripts. **b** 271 repressed transcripts

To identify Arabidopsis candidate genes for response to RSV infection, four transcriptome profiles were analyzed. First, the expression level of each gene was normalized as clean reads per kilobase of exon model per million mapped reads (RPKM). Then, the DEGs were determined by comparing gene expressed in RSV-infected plant samples with those from mock plants at two time points with the stringent criteria of FDR < 0.001 and/log2Foldchange/>1. We obtained 624 DEGs in response to RSV infection at 14 and 21 dpi. At 14 dpi, 255 transcripts were induced, and 38 were repressed by RSV; at 21 dpi, 146 were induced, and 237 were repressed (Additional file 2, Additional file 3). A comparison between DEGs at 14 dpi and 21 dpi identified only 13 genes induced and 4 genes repressed at both of the two time points (Fig. 2). The analysis also revealed that during early symptom development (14 dpi) the number of induced transcripts was greater than repressed transcripts, while later in the infection (21 dpi) repressed transcripts were the predominant DGEs. These results indicated that the majority of genes in response to RSV were unique to a particular time point during infection.

### Functional classification of DEGs in RSV-infected Arabidopsis

**Fig. 3:**
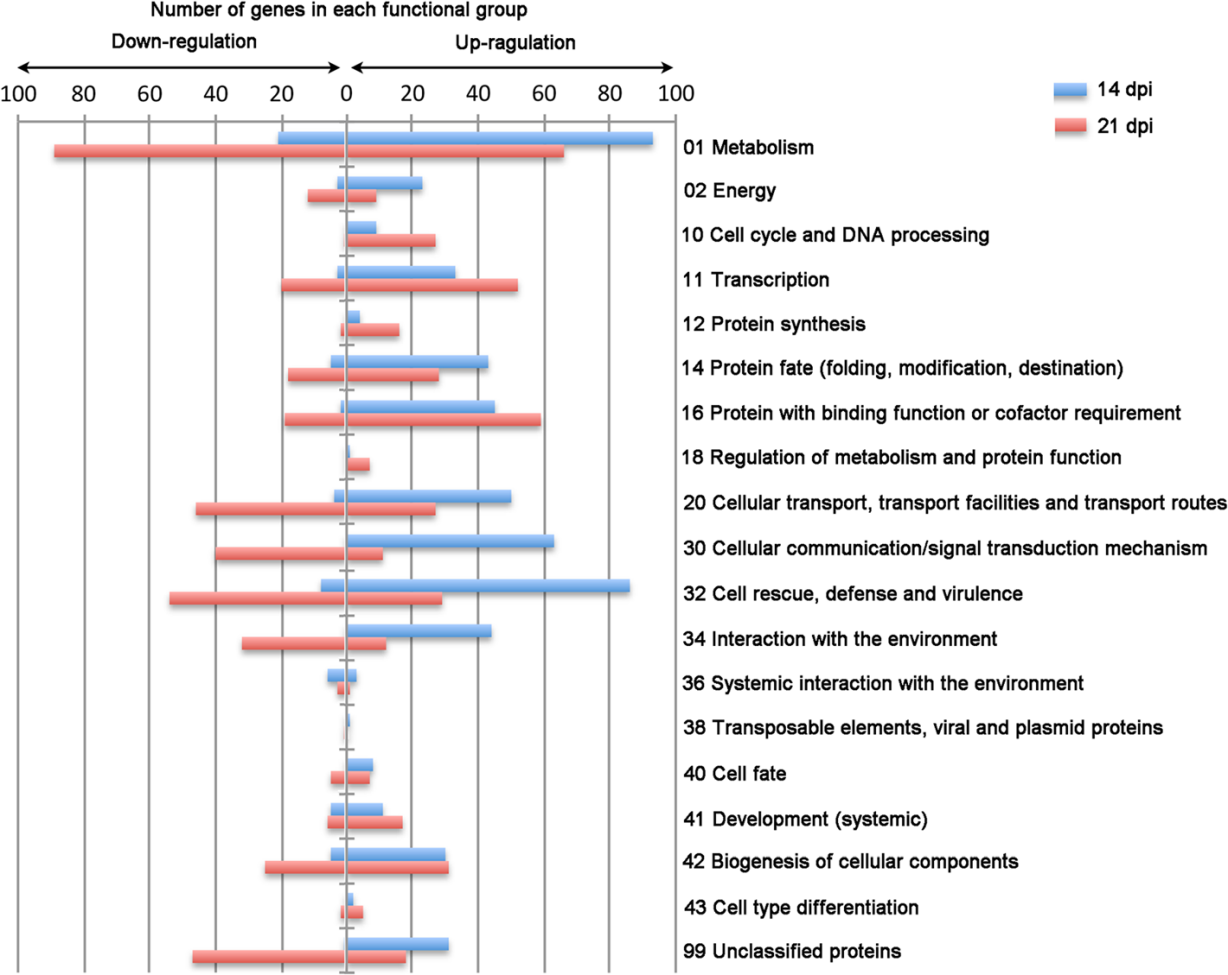
Functional distribution of DEGs in RSV-infected Arabidopsis plants at 14 and 21 dpi

A total of 624 DEGs between RSV-infected and mock treatments were assigned to functional categories following the Arabidopsis MIPS (Munich Information Centre for Protein Sequence) functional classification scheme (Fig. 3). Based on their putative functions, the DEGs were classified into 18 categories associated with metabolism, energy, cell cycle and DNA processing; transcription; protein synthesis; protein fate (folding, modification, destination); protein binding with binding function or cofactor requirement; regulation of metabolism and protein function; cellular transport; transport facilities and transport routes; cellular communication/signal transduction; cell rescue, defense, and virulence; interaction with the environment, systemic interaction with the environment; transposable elements; viral and plasmid proteins; cell fate; development (systemic); biogenesis of cellular components; and cell type differentiation (Fig. 3).

### Gene Ontology (GO) functional enrichment of DEGs by DAVID

**Fig. 4:**
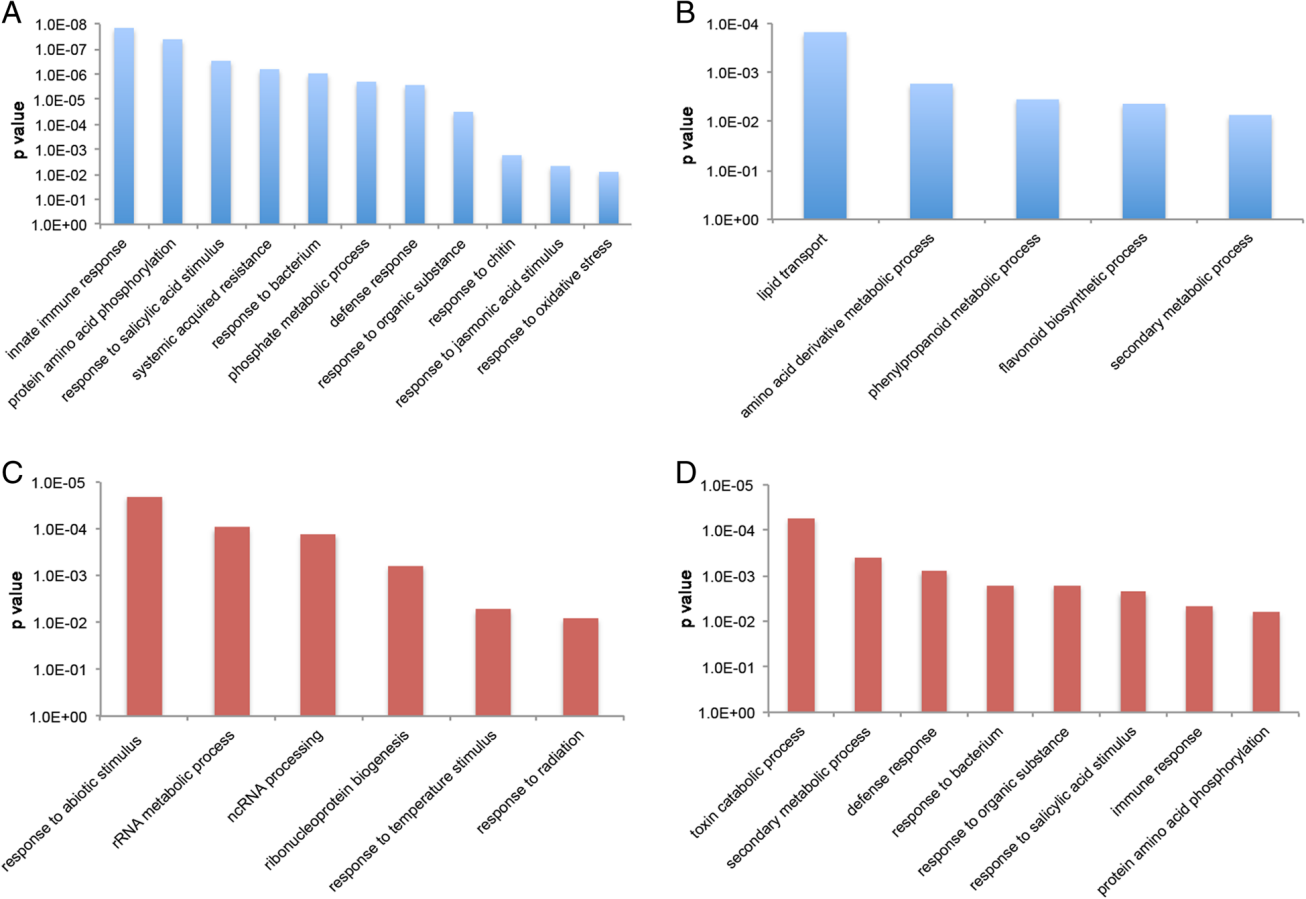
DAVID functional annotation categories of DEGs in RSV-infected Arabidopsis plants. Significantly enriched categories for (**a**) up-regulated genes at 14 dpi; (**b**) down-regulated genes at 14 dpi; (**c**) up-regulated genes at 21 dpi and (**d**) down-regulated genes at 21 dpi

To determine the enriched biological processes in the intimate interaction between RSV and Arabidopsis, the 388 up- and 271 down-regulated genes at the two time points were analyzed using DAVID bioinformatics resources. Among the DAVID functional annotation chart of significantly enriched categories for DEGs induced during the early symptom development stage (14 dpi) were defense response associated processes (innate immune response, response to salicylic acid stimulus, systemic acquired resistance, response to bacterium, response to chitin), protein amino acid phosphorylation, phosphate metabolic process, and response to abiotic stress (organic substance and oxidative) (Fig. 4a). Significantly enriched categories for genes repressed during the early symptom development stage were lipid transport, amino acid derivative metabolic process, and secondary metabolic processes (phenylpropanoid and flavonoid) (Fig. 4b). Later in symptom development (21 dpi), the most significantly enriched categories for induced genes were response to abiotic stimulus (temperature and radiation), rRNA metabolic process, ncRNA processing, ribonucleoprotein biogenesis (Fig. 4c). The significantly enriched categories for repressed genes were toxin catabolic process, secondary metabolic process, defense response associated processes (response to bacterium, response to salicylic acid stimulus, innate immune response), response to organic substance, and protein amino acid phosphorylation (Fig. 4d).

### Identification of DEGs involved in defense signaling in Arabidopsis

**Fig. 5:**
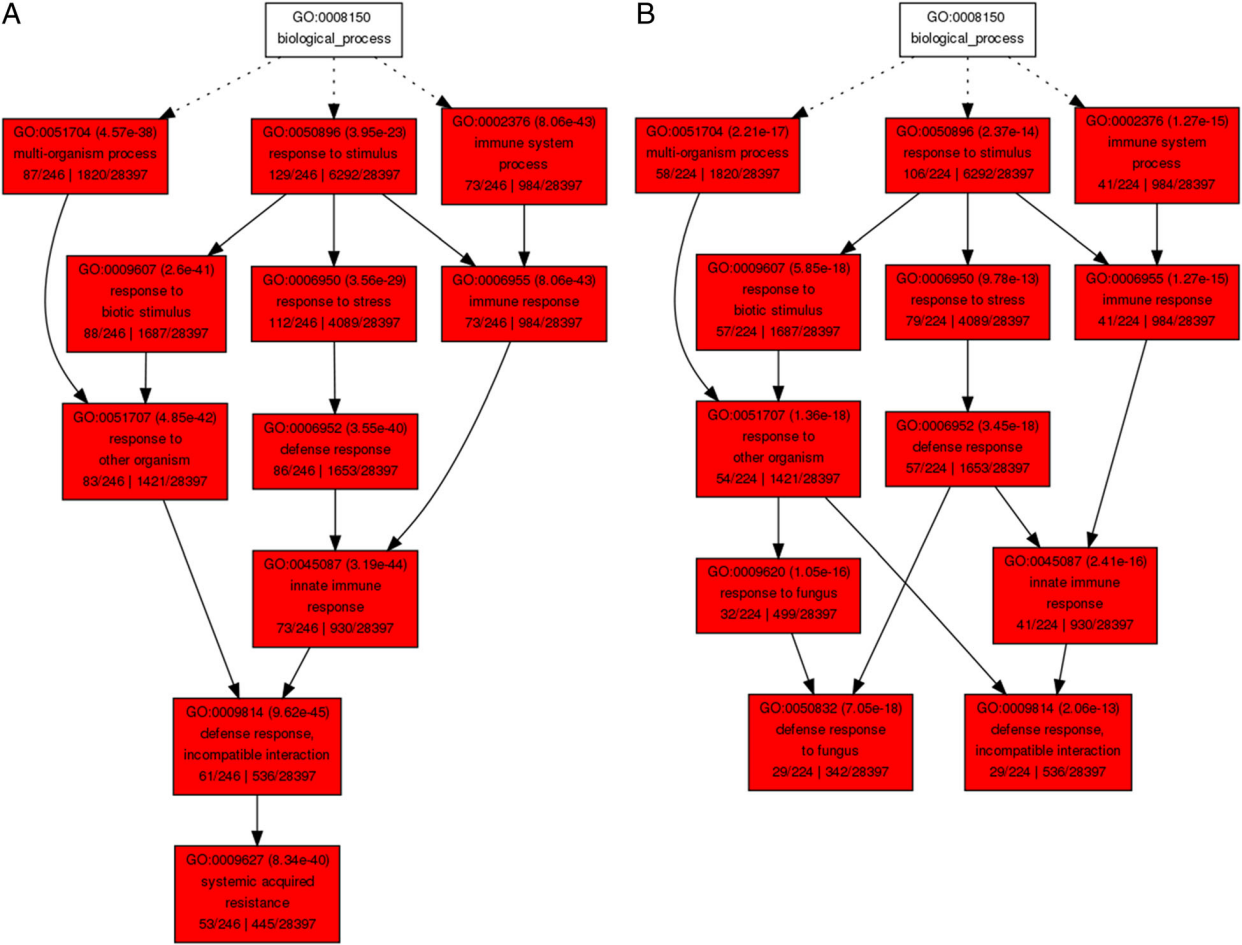
Singular enrichment analysis (SEA) of the DEGs involved in defense response processes at 14 dpi (**a**) and 21 dpi (**b**) using agriGO

GO term enrichment analysis of DEGs by DAVID revealed that RSV up-regulated Arabidopsis defense response gene transcription during the early symptom development stage (14 dpi); however, during the late symptom development stage (21 dpi), most of the defense response genes transcription were repressed by RSV (Fig. 4). The DEGs related to defense response were particularly significance in the agriGO singular enrichment analysis. According to agriGO analysis of DEGs, during early stages of symptom development (14 dpi), among the 255 induced DEGs, 86 (33.7%) were involved in defense response. At the later stage of infection (21 dpi) 57 (24.0%) down-regulated defense-related DEGs were identified among the 237 repressed DEGs (Fig. 5). DEGs modified by RSV infection at these two time points included those with known functions in defense, such as PRs (pathogenesis-related proteins), the disease resistance protein family, kinases, TFs (transcription factors), and salicylic acid mediated signaling pathway proteins. (Additional file 4 and Additional file 5). Among these defense-related transcripts, *GST11* (glutathione transferase 11), *PR1* (pathogenesis-related 1), *CRK36* (cysteine-rich receptor-like protein kinase 36), *AT4g03450* (ankyrin repeat family protein), *WAK1* (cell wall-associated kinase), *AT5g10760* (EDS1-dependent 1), *AT5g45000* (disease resistance TIR-NBS-LRR protein) were up-regulated at 14 dpi but down-regulated at 21 dpi. These data suggest that during early stages of symptom development, Arabidopsis plants respond to RSV infection by expressing defense related genes. When RSV accumulates during later stages of infection, the immune response in Arabidopsis plants is suppressed through an unknown mechanism. Our findings support previous idea that in the compatible interaction between RNA viruses and plants, the suppression of host transcriptional defense responses is a prerequisite for symptom development [1, 2].

### Identification of DEGs involved in secondary metabolism and protein amino acid phosphorylation

Analysis of DEGs by DAVID also revealed that secondary metabolism and protein amino acid phosphorylation were significantly enriched functions. Secondary metabolism plays an important role in defense against herbivores, pests, and pathogens in plants [28]. In this study, DEGs associated with anthocyanins, flavonoids, phenylpropanoids and pigments were down-regulated at 14 dpi and aromatic compound biosynthetic genes were repressed at 21 dpi (Additional file 6, Additional file 7).

Protein kinase cascades are required for salicylic acid (SA)- and jasmonate (JA)-dependent defense against pathogens in plants [29, 30]. DEGs involved in protein amino acid phosphorylation processes such as cysteine-rich receptor-like protein kinases, cell wall-associated kinases, and leucine-rich repeat transmembrane protein kinases were induced at 14 dpi but repressed at 21 dpi (Additional file 8, Additional file 9).

### Identification of RSV induced or repressed genes associated with symptom development

**Fig. 6:**
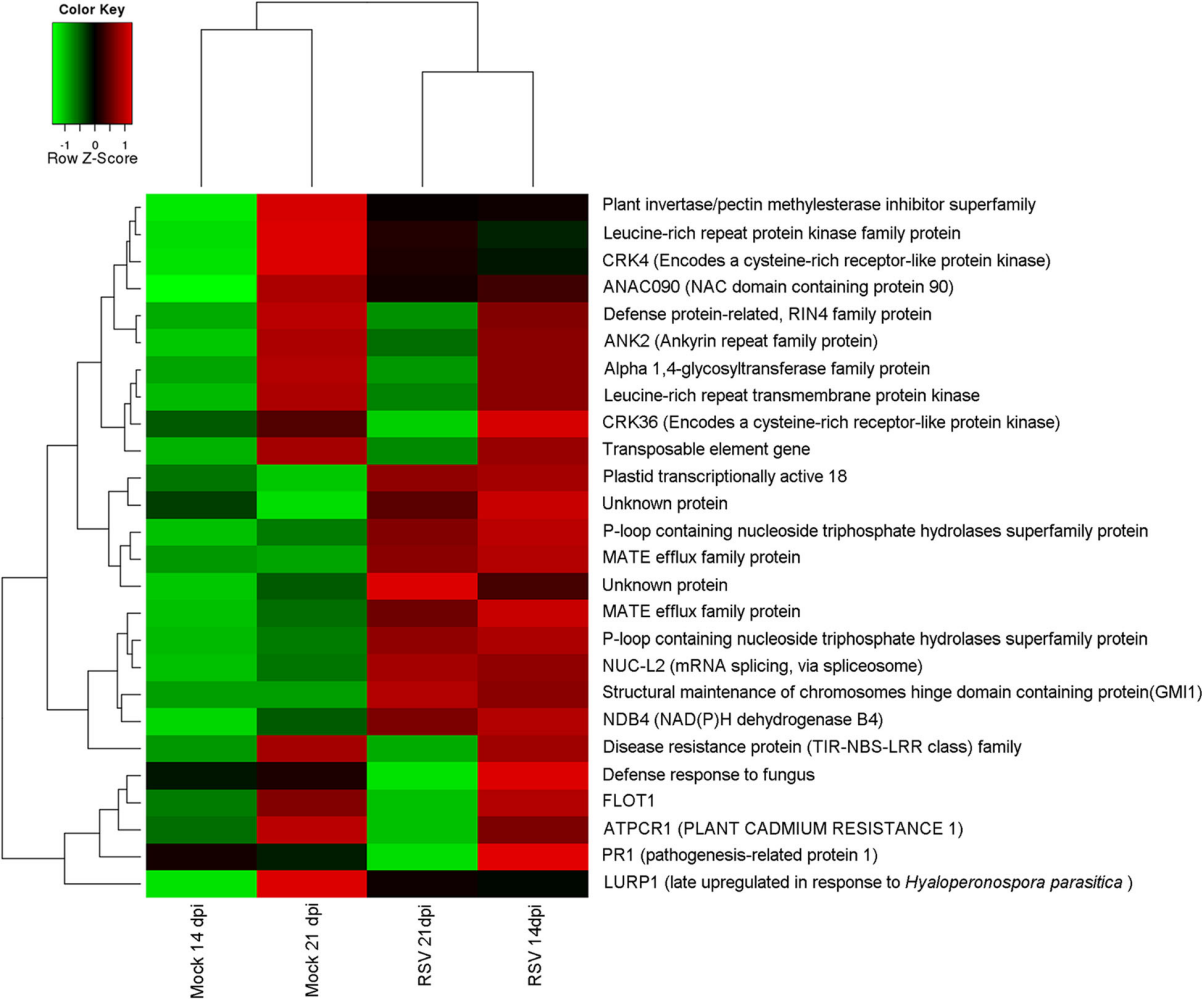
Heat map showing hierarchical clustering of 26 transcripts differentially expressed during both time points (14 and 21 dpi). *Red* bars indicate induction (>2.0), and *green* bars indicate repression (<2.0)

We identified a total of 52 genes that were differentially expressed between the two time points (Additional file 10). By using a 2.0-fold increase or decrease in signal intensity as a cut-off, 26 genes were selected and used to build a heat map (Table 2, Fig. 6). At 14 dpi, all genes were induced by RSV infection; at 21 dpi, 10 genes were induced, and 16 were repressed. These DEGs were shown to be primarily involved in defense responses, protein phosphorylation, transcription, transport and other metabolic processes. These results also indicated that genes selectively induced during the early stage of symptom development by RSV infection, were associated with protein phosphorylation and related defense responses, and at later stages of symptom development the induced genes were involved in metabolic processes such as transport and structural-maintenance.

**Table 2:** DEGs (fold change >2) of 26 transcripts differentially expressed during both time points after RSV infection (14 and 21 dpi)

ATG ID	Description	14 dpi Fold Change	14 dpi Adjusted *P*-Value	21 dpi Fold Change	21 dpi Adjusted *P*-Value
AT1G14880	ATPCR1 (PLANT CADMIUM RESISTANCE 1)	2.52	5.01E-04	−4.14	8.65E-08
AT1G21520	Unknown protein	2.58	2.43E-03	3.11	2.74E-03
AT1G56120	Leucine-rich repeat transmembrane protein kinase	2.19	1.09E-02	−2.04	1.33E-02
AT2G04050	MATE efflux family protein	4.39	4.26E-06	4.12	2.99E-05
AT2G04070	MATE efflux family protein	5.28	2.87E-05	3.03	6.80E-03
AT2G14560	LURP1 (late up-regulated in response to *Hyaloperonospora parasitica*)	2.55	2.36E-03	−2.36	8.67E-03
AT2G14610	PR1 (pathogenesis-related protein 1)	2.58	6.51E-04	−2.38	2.92E-02
AT2G18190	P-loop containing nucleoside triphosphate hydrolases superfamily protein	6.14	1.11E-04	4.62	5.20E-04
AT2G18193	P-loop containing nucleoside triphosphate hydrolases superfamily protein	5.85	3.40E-09	3.90	7.79E-06
AT2G18690	Defense response to fungus	2.02	1.40E-02	−2.14	3.94E-03
AT2G20800	NDB4 (NAD(P)H dehydrogenase B4)	5.14	1.45E-03	2.73	3.27E-02
AT2G26440	PME12 (PECTIN METHYLESTERASE 12)	2.63	6.76E-03	−2.20	3.04E-03
AT2G27402	Unknown protein	2.71	5.24E-04	3.28	9.08E-05
AT3G09020	Alpha 1,4-glycosyltransferase family protein	2.22	2.74E-02	−2.41	1.37E-02
AT3G15357	Unknown protein	2.59	3.29E-02	2.90	4.33E-03
AT3G18610	NUC-L2 (mRNA splicing, via spliceosome)	4.81	3.59E-04	3.97	5.10E-04
AT3G45860	CRK4 (Encodes a cysteine-rich receptor-like protein kinase)	2.95	6.97E-03	−2.62	3.48E-04
AT4G03450	ANK2 (Ankyrin repeat family protein)	2.82	5.93E-04	−2.39	1.86E-03
AT4G04490	CRK36 (Encodes a cysteine-rich receptor-like protein kinase)	2.34	7.40E-03	−2.28	2.24E-02
AT4G06477	Transposable_element_gene	2.57	4.48E-03	−2.30	8.24E-03
AT5G22380	ANAC090 (NAC domain containing protein 90)	4.31	4.58E-04	−2.04	9.01E-03
AT5G24280	Structural-maintenance-of-chromosomes-hinge domain-containing protein (GMI1)	2.29	1.36E-02	2.62	4.56E-03
AT5G25250	FLOT1	2.40	8.17E-04	−2.54	5.16E-04
AT5G45000	Disease resistance protein (TIR-NBS-LRR class) family	3.59	3.29E-02	−3.92	2.50E-02
AT5G48657	Defense protein-related protein	2.27	1.76E-02	−2.53	8.60E-03
AT5G59670	Leucine-rich repeat protein kinase family protein	2.05	2.24E-02	−2.06	3.77E-03

### Confirmation of RNA-seq data by quantitative reverse-transcription PCR (qRT-PCR)

**Fig. 7:**
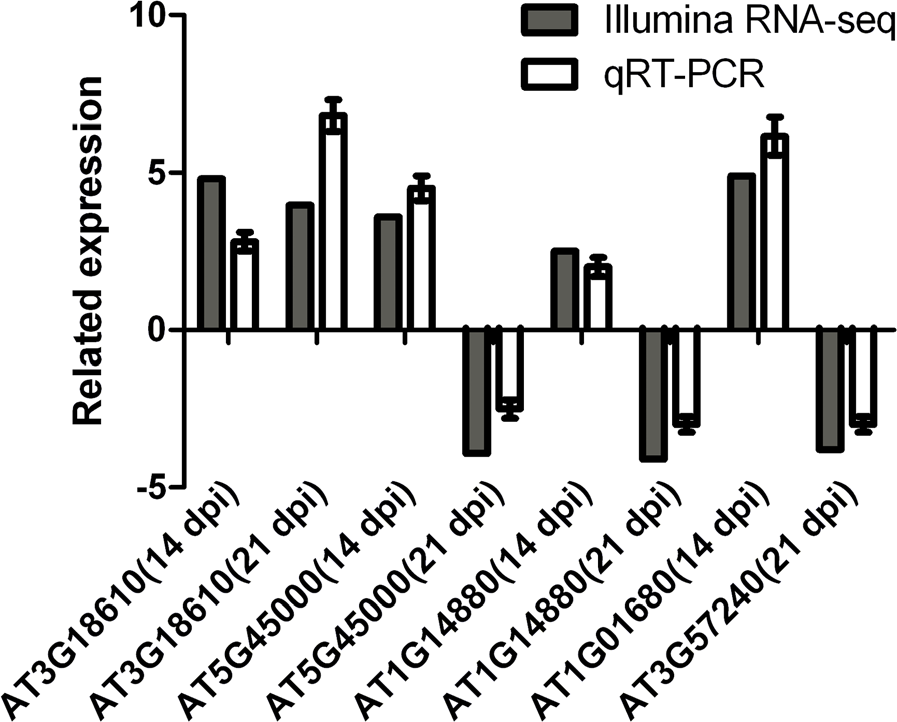
Validation of Illumina RNA-seq expression data by quantitative reverse-transcription RT-PCR (qRT-PCR). Expression patterns selected transcripts that were similar between the two technologies are shown. Signal intensities for each transcript were normalized with *EF1-α* and *actin2*. The x-axis shows the validated genes at 14 and 21 dpi. The y-axis is the normalized fold-change expression values for each transcript

To verify the RNA-seq data, quantitative reverse-transcription PCR was used (Fig. 7). Genes were chose from the 14 and 21 dpi time points. At 14 dpi, four up-regulated genes *NUC-L2* (AT3G18610), *AT5G45000, ATPCR1* (AT1G14880) and *ATPUB54* (AT1G01680) were selected to confirm the expression results obtained from the RNA-seq data. The induced gene *NUC-L2* (AT3G18610) and three repressed gene, *AT5G45000*, *ATPCR1* (AT1G14880) and *ATBG3* (AT3G57240) showed similarities to RNA-seq data at 21 dpi. The results shown in Fig. 7 indicated that all of the gene expression patterns from qRT-PCR were consistent with those from the RNA-seq analysis.

## Discussion

In *Arabidopsis* inoculated with RSV, symptoms started to appear at 14 dpi, and plants were fully symptomatic at 21 dpi. Infected plants showed pronounce stunting and vein chlorosis in the newly emerged leaves (Fig. 1a). The severe symptoms in RSV-infected *A. thaliana* plants suggested that RSV might manipulate and recruit host metabolites for it genome translation and replication like other plant virus [16]. An increase in RSV accumulation in *A. thaliana* plants was observed between time points 14 and 21 dpi showing a 2-fold increase (Fig. 1c, d), confirming that RSV was persistently replicating in *Arabidopsis* leaf tissues and an increase in viral titer associated with disease symptom development. These findings were also observed in rice plants infected with RSV whereby the concentration of CP increased continuously from 9 dpi to 15 dpi [17]. In this study, the transcriptome of RSV-infected *Arabidopsis* plants was profiled. Gene expression data revealed 624 significantly (*p* < 0.05) DEGs (including up- and down-regulated transcripts) in response to RSV infection at two different time points (14 and 21 dpi). Many DEGs were expressed at only one of the two time points. Only a few genes (52, 8.3%) were differentially expressed at both time points during RSV infection, in agreement with our results; previous RNA-seq studies identified 14,381 rice DEGs that responded to RSV infection at three time points but only 532 genes (3.7%) were differentially expressed at all three time points [18]. Together, these data indicate that RSV selectively modifies host gene expression during different stages of viral symptom development.

Postinova and Nemchinov [6] summarized plant general transcriptome responses in compatible interactions between Arabidopsis and eleven viruses (9 RNA; 1 dsDNA; 1 ssDNA) using comparative microarray data. They demonstrated that, in total, the expression levels of 7639 unique genes were significantly changed due to infection by these viruses, and 198 genes were differentially expressed during all eleven virus infections. Compared with these results, RSV shared 279 (across two time points) in common with the 7639 unique genes (Additional file 11), only 16 genes were in common with the 198 genes (Additional file 12), indicative of the unique characteristics of each virus-host interaction. Among the small pool of genes that were regulated by RSV and these other viruses, many genes were involved in defense responses, responses to biotic stimulus, and cellular amino acid and related metabolic processes. Among these defense genes, ß-1,3-glucanase (AT3G57260) was shown to be up-regulated at early stages of infection by RSV (14 dpi) and other RNA viruses (TVCV, ORMV, PVX, CMV, and TuMV at 2, 4, and 5 dpi) [1]. In previous studies, degradation of callose by ß-1,3-glucanase increases the plasmodesmata (Pd) size exclusion limit (SEL) and facilitates cell-to-cell movement of RNA viruses [31, 32]. This indicates that defense responses and Pd gate modification mechanisms are generally conserved plant responses to RNA viruses [33, 34].

In susceptible plants, viral infections result in activation of the small RNA silencing antiviral machinery and plant hormone signaling defense pathways [35, 36]. The results of this study suggest that genes participating in RNA silencing pathways may not be activated in RSV-infected Arabidopsis plants during the symptom development. These results may be explained by the fact that RSV encodes two gene silencing suppressors (NS2, NS3) that inhibit local and systemic gene silencing [15, 37]. In contrast, in rice plants RSV activates the gene silencing system during late stages of infection. Some rice genes belonging to the Argonaut protein family, such as *OsAGO1a*, *OsAGO1b*, *OsAGO1c*, *OsAGO12* and *OsAGO18*, are significantly up-regulated by RSV, but the transcript levels of genes encoding DICER-like and RDR proteins were not changed [18, 38]. These dissimilarity may be caused by different host plants, Arabidopsis, an experimental host of RSV and *O.sativa*, a natural host of RSV.AGO12 and AGO18 proteins have been found only in grass genomes, but not flowering plants such as Arabidopsis [39]. Additionally, the comparative analysis of RSV-derived vsiRNA from *O. sativa* and *N. benthamiana* (another experimental host), revealed that the number and size distributions of vsiRNAs in the two hosts were very different [40]. These data demonstrate that RSV has host-dependent effects on the expression of genes involved in RNA silencing pathways. It should be noted that because this study has only examined Arabidopsis plants with viral symptom expression (14 dpi and 21 dpi), we cannot rule out the possibility that the transcripts of RNA silencing pathway genes would change at early stage of RSV infection. Thus, the functional roles of RNA silencing associated with this virus should be investigated in future experiments.

Activation or suppression of plant hormone signaling defense pathways is a common response to infection with RNA viruses, DNA viruses, and viroids in several different plants. Plants mostly activate salicylic acid (SA)-signaling and jasmonic acid (JA)/ethylene (ET)-signaling pathways, which are regulated antagonistically by each other, against various pathogens [41, 42]. Salicylic acid signaling plays a crucial role in the defense against biotrophy, whilst the defense responses against necrotrophic pathogens is mediated by the jasmonic acid/ethylene signaling pathway [41, 42]. The results of this study indicate that the genes related to salicylic acid synthesis, PR proteins, gluthation S-transferase (GST), and other defense-related proteins were up-regulated by RSV infection at the early stage (14 dpi), but were suppressed at the later stage (21 dpi). Among these defense-related proteins, cysteine-rich receptor-like kinase 36 (CRK36) (At4g04490) plays important role in innate immunity, as overexpression of CRK36 in Arabidopsis increased resistance to bacteria [43]. Patatin-like protein 2 (PLP2, At2G26560) encodes a lipid acyl hydrolase, promotes cell death and contributes to resistance to *Cucumber mosaic virus* [44]. The defense-related gene expression profiles in Arabidopsis during RSV infection imply that at later stages of infection when virus accumulation increased and disease symptom developped led to suppression of plant defense systems, which is in agreement with studies of other plant-virus combinations [1, 2]. In rice plants, the transcription of defense genes was strongly affected by RSV infection, and the number of up-regulated defense genes was higher than that of the down-regulated defense genes [18]. Although, there is seemingly some host-dependent variation in the expression patterns of defense genes during RSV infection, we suspect that these defense pathways might be especially important in plants during interaction with RSV.

We identified individual gene transcripts during two time points, and some overlap of transcripts was also observed between the time points (Fig. 2). Persistent expression of transcripts (during both time points) may be necessary to carry out functions associate with defense responses to resist virus attack or aid in viral replication, cell-to-cell spread or systemic movement, as implicated in other studies [1, 2]. Only 26 transcripts with were identified during both time points in RSV-infected *Arabidopsis* (Table 2), indicating that most genes were transiently expressed and not sustained during the infection. Examples of these transcripts include: LURP1 (AT2G14560), which is required for basal resistance to *Hyaloperonospora parasitica* and is induced by salicylic acid and *oilseed rape mosaic virus* (ORMV) [45, 46]; and PME12 (AT2G26440), which encodes a pectin methylesterase that is important for immune responses against the necrotrophic fungal pathogen *Botrytis cinerea* and the bacterial hemibiotroph *Pseudomonas syringae* [47]. Another interesting gene up-regulated at 14 dpi but down-regulated at 21 dpi by RSV encodes a disease resistance protein, TIR-NBS-LRR (toll-interleukin-1-receptor/nucleotide-binding site/leucine-rich repeat). In the Arabidopsis genome, there are 94 TIR-NBS-LRR genes, which comprise the largest class of plant disease resistance genes [48]. In the Arabidopsis Est ecotype, TTR1 encodes a TIR-NBS-LRR protein that controls the ecotype-dependent resistance to *Tobacco ringspot virus* (TRSV) [49]. It would be interesting to find out whether the TIR-NBS-LRR genes play an important role in plant defense against RSV infection.

## Conclusions

A large number of Arabidopsis genes that are differentially expressed during RSV infection at two time points were identified by DGE analysis. These DEGs were associated with multiple biological functions, including defense responses, secondary metabolism, protein amino acid phosphorylation and responses to abiotic stress. Importantly, we also showed that at early (14 dpi) and late (21 dpi) stages of viral symptom development during RSV infection, a total of 52 DEGs are differentially expressed between these two time points. GO term analysis, in a RSV-Arabidopsis compatible interaction, indicated that basal defenses are induced but are not capable of inhibiting viral replication and movement at early stages of viral symptom development. During the infection period, the suppression of host defense responses may be associated with disease symptom severity. Differences of DEGs between Arabidopsis and rice plants during RSV infection may in part reflect different adaptations and evolutionary paths of the virus and host plants. This study provided additional insights into the molecular basis of Arabidopsis responses to RSV infection. Functional characterization of candidate genes through overexpression and reverse genetics approaches is required to better understand RSV-host interactions.

## Additional files

Additional file 1: Table S1. Primers sequences used for the validation of DEGs and expression of RSV CP and SP genes. (DOC 33 kb)
Additional file 2: Table S2. DEGs expressed at 14 dpi. (XLSX 73 kb)
Additional file 3: Table S3. DEGs expressed at 21 dpi. (XLSX 78 kb)
Additional file 4: Table S4. Up-regulated DEGs involved in defense response at 14 dpi. (XLSX 56 kb)
Additional file 5: Table S5. Down-regulated DEGs involved in defense response at 21 dpi. (XLSX 54 kb)
Additional file 6: Table S6. DEGs involved in secondary metabolism at 14 dpi. (XLSX 51 kb)
Additional file 7: Table S7. DEGs involved in secondary metabolism at 21 dpi. (XLSX 55 kb)
Additional file 8: Table S8. DEGs involved in protein phosphorylation at 14 dpi. (XLSX 52 kb)
Additional file 9: Table S9. DEGs involved in protein phosphorylation at 21 dpi. (XLSX 52 kb)
Additional file 10: Table S10. DEGs involved in protein phosphorylation at 21 dpi. (XLS 17 kb)
Additional file 11: Table S11. A comparison of DEGs between RSV and eleven plant viruses in Arabidopsis. (XLSX 36 kb)
Additional file 12: Table S12. DEGs affected by RSV and all eleven viruses. (XLSX 81 kb)

## Abbreviations

DAVID: Database for annotation, visualization and integrated discovery
DEGs: Differentially expressed genes
DGE: RNA-seq based digital gene expression
Dpi: Days post-infection
ET: Ethylene
GO: Gene ontology
JA: Jasmonic acid
ORFs: Open reading frames
qRT-PCR: Quantitative reverse-transcription PCR
RdRp: RNA-dependent RNA polymerase
RPKM: Reads per million mapped reads
RSV: Rice stripe virus
SA: Salicylic acid
SEA: Singular enrichment analysis
TIR-NBS-LRR: Toll-interleukin-1-receptor/nucleotide-binding site/leucine-rich repeat
TRSV: Tobacco ringspot virus
