# Supplementary material for: RNA-seq-based digital gene expression analysis reveals modification of host defense responses by rice stripe virus during disease symptom development in Arabidopsis
Source: Virol J. 2016 Dec 2;13:202. doi: 10.1186/s12985-016-0663-7 (PMC5134058; doi:10.1186/s12985-016-0663-7)
Supplement: Additional file 1: Table S1. — Primers sequences used for the validation of DEGs and expression of RSV CP and SP genes. (DOC 33 kb) [file 12985_2016_663_MOESM1_ESM.doc]

Table S1. Primers sequences used for the validation of DEGs and expression of RSV CP and SP genes.

| Gene | Primers | Product Size (bp) | tests |
| --- | --- | --- | --- |
| *CP* | 5’-AGGATGTGACAACTTACTGTGGGACT-3’  5’-GACTTGCATGTGATGACCAGGAGA-3’ | 237 | qRT-PCR |
| *SP* | 5’-TTGTCACTCATTCTTATCACACCTG-3’  5’-TTCTTCCACACTTTCTCATACTCTT-3’ | 237 | qRT-PCR |
| *AT3G18610* | 5’-GTACTCCACGAAACAGCAATCCTG-3’  5’-TGAACCCTTGTCACCTCTCCACAC-3’ | 154 | qRT-PCR |
| *AT5G45000* | 5’-ATAGGAAGTGGACAAAAACAAGA-3’  5’-TCAAACACCGATATGACTCAGAAT-3’ | 103 | qRT-PCR |
| *AT1G01680* | 5’-TCCATTTTCGTTGACCACTTCT-3’  5’-ATCTTTTTCATTAACCCCTTTG-3’ | 142 | qRT-PCR |
| *AT1G14880* | 5’-GAGATGGTTGTACTGATTGCCT-3’  5’-CTCCACCTTGATTTTGTTGTTT-3’ | 144 | qRT-PCR |
| *AT3G57240* | 5’-TTACCACTGTTATTGCTTCTTCTCA-3’  5’-GGTCGCAGGTTGTTTCCATTTCTCC-3’ | 107 | qRT-PCR |
| *EF1-a* | 5’-GGCTGCTGAGATGAACAA-3’  5’-GTGGTGGAGTCAATGATAAG-3’ | 225 | qRT-PCR |
| *Actin2* | 5’-GCAAGTCATCACGATTGGTGC-3’  5’-GCAACGACCTTAATCTTCATGCTG-3’ | 156 | qRT-PCR |
